# How Competition for Funding Impacts Scientific Practice: Building Pre-fab Houses but no Cathedrals

**DOI:** 10.1007/s11948-024-00465-5

**Published:** 2024-02-13

**Authors:** Stephanie Meirmans

**Affiliations:** Department of Ethics, Law and Humanities, Amsterdam UMC, Academic Medical Center, University of Amsterdam, Meibergdreef 9, Amsterdam, 1105 AZ Netherlands

**Keywords:** Ethnographic, Questionable research practices, Research integrity, Science in practice, Projectification, Risky science

## Abstract

In the research integrity literature, funding plays two different roles: it is thought to elevate questionable research practices (QRPs) due to perverse incentives, and it is a potential actor to incentivize research integrity standards. Recent studies, asking funders, have emphasized the importance of the latter. However, the perspective of active researchers on the impact of competitive research funding on science has not been explored yet. Here, I address this issue by conducting a series of group sessions with researchers in two different countries with different degrees of competition for funding, from three scientific fields (medical sciences, natural sciences, humanities), and in two different career stages (permanent versus temporary employment). Researchers across all groups experienced that competition for funding shapes science, with many unintended negative consequences. Intriguingly, these consequences had little to do with the type of QRPs typically being presented in the research integrity literature. Instead, the researchers pointed out that funding could result in predictable, fashionable, short-sighted, and overpromising science. This was seen as highly problematic: scientists experienced that the ‘projectification’ of science makes it more and more difficult to do any science of real importance: plunging into the unknown or addressing big issues that need a long-term horizon to mature. They also problematized unintended negative effects from collaboration and strategizing. I suggest it may be time to move away from a focus on QRPs in connection with funding, and rather address the real problems. Such a shift may then call for entirely different types of policy actions.

## Introduction

There seems to be a crisis in science: surveys have recently found that many researchers engage in so-called questionable research practices (QRPs) (Bouter et al., 2016; Kaiser et al., 2021; Xie et al., 2021; Gopalakrishna et al., 2022). For example, they submit to selective reporting, p-hacking, and HARK-ing in order to score good publications (Bouter, 2020, following Wichters et al., 2016). Though these QRPs are typically viewed as less problematic than research misconduct cases (FFP: fabrication, falsification, plagiarism), they have been found to be more widespread (Steneck, 2006; Fanelli, 2009, 2010). Bouter et al. (2016) therefore made the case that QRPs might do more harm to science than the less frequent misconduct (FFP) cases. For the remainder of the paper, I will use the concepts of QRP and research misconduct as used in this 2016 paper. Many research integrity scholars assume that it is the increasingly competitive nature of science, and in particular the need for high-impact publications and funding, which may be the main driver for individual researchers resorting to QRPs (Martinson et al., 2005, 2006; Bouter, 2020; but see Fanelli et al., 2015).

While for a long time there has been a focus on the individual researcher behaving badly, the focus in the research integrity debate has in recent years shifted away from individual responsibilities (and spectacular cases of fraud) towards aspects of scientific communities and research climate. Therefore, Zwart and ter Meulen (2019) have urged to investigate how universities and funders could help fostering a culture of research integrity.

Remarkably, funding thus seems to play two contradictory roles in the discussion around research integrity: on the one hand, competition among researchers for limited funding is seen as potentially elevating QRPs. On the other hand, funders are also seen as potential agents to foster research integrity. So how does this actually play out in practice?

Labib et al. (2021; see also Mejlgaard et al., 2020) have recently made a first step in investigating how funders think they could help fostering research integrity. Labib et al. (2021) established eleven themes from the RI literature with regards to funding, and then asked the funders about the significance of each theme using surveys. The top three themes to enhance responsible science that emerged were “dealing with breaches of RI, conflicts of interest, and setting expectations on RPO’s (= research performing organizations)” (Labib et al., 2021). Funders were thus seen as being able to impose requirements on research organizations, such as universities, with regards to the implementation of research integrity measures (see also Roje et al., 2021). In addition, some funding agencies foster research integrity by requiring mandatory online RI training (e.g. EMBO in Europe, NIH and NSF in the USA).

What is currently lacking in the debate is the perspective from active researchers: how do researchers themselves experience the impact of competition for funding on QRPs? And how does the degree of competition factor into this? In this study, I investigate how active researchers experience the impact of competitive funding on their research, in a high versus in a low competitive setting.

## Methods

### Pilot Study

Initially, I wanted to test the following hypothesis: does competitive research funding increase QRPs as defined by Bouter et al. (2016)? If so, one should find that there are more QRPs reported by researchers working in a country with a high competition level than those in a country with low competition. To optimize the questions asked in researcher group sessions, I first conducted a pilot study consisting of a series of single interviews with researchers from various scientific fields. From the first couple of these pilot single interviews, mostly conducted in Switzerland in 2017, it quickly became clear that many researchers did not see a direct connection between competitive research funding and what are called QRPs. This did not mean, however, that my interviewees did not see any questionable effects of competitive research funding on doing good science. My interviewees told me many, and apparently for them quite serious, problems. However, these problems were often of a quite different nature than what is typically being captured under QRPs in the research integrity literature. I had also introduced my interviewees to the main QRPs found in the 2016 paper by Bouter and colleagues, but they typically did not see a direct connection to funding.

In addition, it became clear that not all QRPs are applicable to all fields. This was recently confirmed in a large-scale survey study, performed after my study ended, in which humanities scholars attested ‘not applicable’ to a large range of QRPs (Gopalakrishna et al., 2022). My interviewees also told me that it can even be the case that what is called a QRP in one discipline can be a responsible practice in another (see also Ravn & Sørensen, 2021 for a similar recent finding): for example, diverging from an original research question is a virtue in the humanities but a vice in a medical study. My pilot study thus indicated that there was a serious problem with the original research design of my study: the overly narrow definition of QRPs made it difficult to obtain a universal understanding of the effects of competition for research funding on questionable/ responsible research practices.

Due to the insights gained during these pilot interviews, I decided to shift the original research question in the follow-up group sessions to a more open but rather simple question: “How does competitive research funding affect science (in good or bad ways)?” This question was accompanied by a follow-up question on what could be done better (results will follow in another publication).

### Study Design

The research question was addressed using an experimental design involving group sessions with active researchers in two different countries: one country with a high level of competition for research funding, and one country with a low level of competition for research funding. The Netherlands was chosen as the ‘high-competition’ country (with grant success rates of 20–30%), and Switzerland as representative of a relatively ‘low-competition’ country (grant success rate 50–60%) (according to the Rathenau Institute and the Swiss Science Council respectively, personal communication). Both countries were also chosen out of convenience: the Netherlands was the country in which this study was based, Switzerland was chosen because there were pre-existing ties that enabled an efficient set-up. The study design included a comparison across different disciplines (natural sciences, medical sciences, humanities) and ‘seniorities’ in career stage – the idea being that juniors might be under higher pressure to obtain funding.

### Participant Selection and Group Session Details

In each country, six group session interviews were conducted in 2018. The groups typically consisted of four or five researchers, with a minimum of three for one session (natural science senior NL), and a maximum of seven researchers for another session (medical sciences senior CH). These researchers were grouped by scientific field (natural sciences, medical sciences, and humanities) and career status. Career status was distinguished as ‘junior’ (= temporary employment) or ‘senior’ (= permanent employment)^1^. This made a total of twelve group sessions with in total 57 persons in a very balanced across-groups design. Participants matching the criteria above were recruited via personal networks as well as via Dutch and Swiss university websites and the website of the Royal Netherlands Academy of Arts and Sciences. I aimed for as much gender balance as possible. I facilitated the sessions together with a colleague who opted out of co-authoring the paper.

I checked session participants for their experience with funding ahead of the sessions and noticed that the recruitment strategy resulted in a high number of experienced researchers with funding. Most senior researchers had received multiple types of funding in the past (both via national funds, but many also had received EU funding, including ERC grants for several participants). Many seniors had additional experience with participating in funding reviewing panels, at both the national and the international level, including for the ERC.

Each session took 3.5 h and took place in person either in Switzerland or in the Netherlands. At the beginning of each session all researchers were familiarized with the same background information of the study (QRP’s, mainly as explicated in Bouter et al., 2016) as well as with the idea that they could also explore other impacts of competition for funding on science, including in a positive sense. Written informed consent was obtained from all session participants. Researchers in the sessions each sat with a laptop or tablet around a round table, and during the sessions made extensive use of a digital tool called “Meetingsphere”. This tool is designed to allow anonymized digital interaction between session group members (https://www.meetingsphere.com). The tool was chosen due to the sensitive nature of the question, allowing honest answers regarding research integrity problems. It also allows a more equal contribution by each group session member, introducing less bias via outspoken members of the group. At least half of the session time was spent on the main research question: ‘How does competitive research funding affect science (in good or bad ways)?’ Group members were first allowed to type their answers into the digital system. When the rate of new answers slowed down (typically after 10–15 min), the system was opened for digital cross-commenting (again around 10 min), followed by extensive oral discussion. One of the groups (Swiss natural science seniors) ended up with oral discussion only due to the late arrival of one of the session participants. My analysis for this paper focused on the digital session reports only and not on the oral discussions; the Swiss natural science group (with four participants) was therefore excluded from the analysis.

### Analysis of Session Reports

I used a grounded thematic analysis (e.g. Charmaz, 2006) in several rounds to analyze the Meetingsphere reports. These reports contained the answers typed by the participants as well as the cross-comments. These reports were not large, typically around 4 pages, making the analysis relatively straightforward. The themes emerged entirely from the content of the Meetingsphere reports. I ran the first round of analysis in parallel with three other researchers. There was a near-complete overlap in themes detected between the four of us. The only disagreement between us regarded the inclusion of a separate major theme around research misbehavior and sloppy science. The number of statements regarding such QRPs seemed too few to some of us to warrant being taken up as a major theme at all. However, given the original purpose of the study it also felt odd to not include this theme, and it was therefore included in the end.

In subsequent rounds, I refined the themes and split them into subthemes. More precisely, I first put all the quotes from each theme into separate Word files, and created subthemes. This analysis was done by hand (with different colors of markers on the printed text). Doing this simply by hand turned out to be the best option due to the limited number of pages and simultaneous high density of information provided in the session reports.

## Results

Session participants were prolific in providing input using the Meetingsphere software, both with regards to initial own answers and in reaction to others’ answers. Via my thematic analysis, I identified a couple of main themes and subthemes that researchers addressed regarding the impact of competitive research funding on science in good or bad ways. The three recognized main themes were: (1) The impact on how science is being shaped due to the competition for funding, (2) The impact of grant writing on research time, (3) The impact of publication pressure on QRPs.

### Shaping Science

By far most comments (262/317) focused on how science is being shaped in practice via funding, and how this influence is being perceived and experienced. Importantly, these impacts are not seen as resulting in essentially wrong or sloppy science. Typically, the impact is experienced due to funder interventions, in both positive and negative ways. What typically happens is that researchers do understand and appreciate that funders select projects based on certain features, and that they intentionally shape funding calls and schemes in particular ways (positive). However, funder interventions can have unintended consequences, and these can then be experienced as problematic by researchers (negative). Below, I provide an overview of the perceived impacts in subthemes. While some subthemes present positive and negative effects in a more balanced way, others show that the effects are predominantly experienced as negative. I also provide the number of comments within each subtheme, to give a sense of how much attention there was for each of the subthemes.

#### Impact on Science via Peer Review

There were many comments (61) on how funder peer review impacts science. Though many researchers stated that competitive research funding should in theory increase overall quality in science, only some researchers –all of them Swiss– thought this is indeed the case in practice.

funding is brought to the best research ideas and best people (nat jun, CH)

One Dutch researcher commented that success in funding acquisition often means future successes in gaining funding as well. This researcher was neutral about the effects on science via such a process: “*I do not know whether it’s good or not.”* (hum jun, NL).

There were a few comments on the positive effects of the competition on research practice. For example, one Swiss medical senior scientist said that *“it improves research quality”*. Researchers across countries and disciplines also expressed that projects that are submitted to funders typically have been thought through and tend to have solid methodologies. The feedback of reviewers can additionally help to improve the research, two Dutch natural senior scientists thought.

However, other –in particular Dutch– researchers perceived that while this is how it should work in theory, the practice looks different. One important problem is that peer-review highly depends on the reviewers and the committee/ panel, and these can be biased towards their own research interests. Many told us that their comments were based on personal experiences, and negative experiences with such biases in peer review led some Dutch senior researchers to state that peer review does not work anymore.

Humanities scholars (in both countries) thought that there is a severe problem because reviewers and panels can be biased if they represent certain research schools or fields. Such biases can even lead to a competition between scientific disciplines:

how to avoid that competition between projects turns into competition between disciplines? (hum sen, CH)

In the Netherlands, there was a specific problem with clustering of social sciences and humanities into one program. Due to differences between the two fields regarding how to recognize good research (e.g. many multi-authored article publications versus a few single-authored books), several humanities scholars felt they had a lower chance to obtain funding. In addition, one researcher noted that some board members from the social sciences rejected non-empirical research. However, this ultimately meant rejection of large areas of humanities research. The same type of bias was thought to play a role in gaining funding for medical qualitative research (where methods are different than in mainstream more quantitative medical research).

in the combined humanities & social-science boards, there is no understanding of what a humanities research project may look like. (hum sen, NL)

On the positive side, one medical senior scientist expressed the view that an alternative system to the competitive research funding system might either not exist or be worse. In addition, several younger and older Swiss and Dutch humanities researchers mentioned that funding/ peer review can also enable young researchers to broaden their research.

Young researchers have the chance to free themselves from their home institutions by applying for funding and thus gain access to other cultures, ways of doing science. (hum jun, CH).

#### Impact on Novel and Risky Science

Researchers across all 11 groups submitted many comments (61) on whether and how funding impacts novelty and risk in science. The comments were predominantly negative. Many expressed that while funders often aim to fund innovative and risky projects, the opposite typically happens. One Dutch researcher commented that the *“rhetoric of innovation and breakthrough”* does not reflect how most funding is awarded in practice (hum jun, NL). The reason for this is that research projects are designed to be funded, not designed towards what researchers themselves would consider to be novel ideas, and to be creative and original science:

in principle, good effort to support the best science, but the measures of success are in favour of ‘productive’ science, not necessarily creative science (med sen, CH)

the competitive system only works for ideas and methodologies that are well established, well known, not for ideas and methodologies that are new and really original (hum sen, NL)

One reason for this is that funders put too much emphasis on the track record of the researcher, meaning that one dares not to stray too far away from one’s own disciplinary grounds and instead plays safe. It “*encourages researchers to take small steps in the development of research ideas instead of taking a larger risk and trying something completely different”* (med jun, CH). It imposes a “*disciplinary straightjacket”* (hum sen, CH) to the individual researcher, it encourages researchers to “*remain within areas in which you have already proven yourself with publications”* (hum sen, CH).

Changing fields is discouraged in the current structure of competitive funding, a characteristic that is not supportive of interdisciplinarity and innovation. (med jun, CH)

For science, this means that research will progress only in “*incremental steps”* (med jun, NL), while this may not be the best research: “*it probably leads to conservative research”* (hum jun, NL). And this, according at least to my interviewees, might in the end be counterproductive to what good science should be all about: taking risks, venturing into the unknown.

#### Impact on Science via Funder Research Agenda

Many researchers across countries experienced that funders steer what kinds of research can be done (40 comments); this can be positive because money can strategically be put into solving important challenges:

It enables society and politics to focus scientific research on key societal challenges and problems. In this sense, it contributes to societal problem-solving. (nat jun, NL)

However, most researchers across countries experienced funder agendas as being problematic because they might not foster the best science. Swiss scientists also commented that it would be problematic if the funder agenda would bias against doing basic research:

Negative/comment: It would be disastrous if competitive funding schemes would push research away from fundamental science (nat jun, CH)

Indeed, many senior Dutch natural scientists experienced just that, even though some also saw positive aspects in more applied ways of doing science.

Negative: nearly all 100% fundamental project funding possibilities in NL are being eliminated. Even the Science Agenda is now funded with contributions from industry. (nat sen, NL)

In general, Dutch researchers experienced that a focus on societal impact can take time away from doing core research work. Funder bias towards societal impact can also mean that bigger research fields or those with a higher applicability are more likely to get funded, which both natural and medical scientists across countries experienced as problematic.

#### Impact on Science via Incentivizing Collaborations

Researchers frequently reported that funding has effects on collaborations (29 comments). Funding typically fosters collaborations, and many researchers regarded this in principle as positive. For example, one Dutch medical senior noted that *“it helps to establish interactions and networks beyond the finally funded projects”.* Another medical Swiss senior expressed that *“the process of writing applications already has major impact on creating innovative idea and collaborations”*.

However, many researchers also experienced that those collaborations often do not work well in research practice. This can be due to a variety of reasons, such as too large consortia, inter-disciplinary problems, or feeling forced to collaborate. This can be problematic to such a degree that collaborations have overall negative effects. Medical seniors frequently uttered such skepticism about large consortia/ interdisciplinary multicenter collaborations. They said that they do not work well, there are communications problems between disciplines, and they would *“need better support and guidance”* (med sen, CH). They can be forced upon you, and lead to a lot of *“formal interaction without actual benefits”* (nat jun, CH).

forming strong consortia to increase chances; this can also be a disadvantage if you feel obliged to cooperate with groups for increasing chances on funding, but that will either just complicate the research process/feasibility or even be a disadvantage (med sen, NL)

This all could lead to dishonesty about collaborations in applications. One senior Swiss humanities researcher wrote that collaborations often exist only on paper. Funding could also lead to confusing effects, for example in the humanities where there is no tradition of ‘team research’.

#### Impact on Science via Research Plannning

Many researchers across groups expressed that applying for funding has a positive effect on thinking through, planning and structuring research (20 comments). This can make researchers *“think about next steps in your research”* (med sen, NL) and think carefully about what to do and how to do it. Ultimately, this *“might help to make [the research] more effective and more fruitful”* (hum jun, NL). Some Dutch natural scientists also expressed that the need to apply for funding could even help to come up with new ideas and trigger new collaborations, for example with other groups with better skills. It also enables the researcher to spend time on thinking and getting up to date with the literature.

Dutch medical senior researchers also perceived that the way good science should be done is often at odds with the way funding requires research to be planned:

It also limits flexibility to change the design when needed or address additional question which appear more interesting on the way. (med sen, NL)

One Dutch natural science researcher thought this is not so much of a problem in practice, because “*surely no one does exactly what is in the grant, right? You write a cool proposal and decide later what’s actually possible”* (nat jun, NL). Other researchers did feel forced to become dishonest in their grant-writing in order to circumvent this epistemic problem:

Bad: Science is per definition not predictable. Competitive funding forces you to predict your science, i.e. first do experiments then write the grant. Afterwards claim success because all your ‘predictions’ turned out to be true. This is often termed ‘pilot’-data (med sen, NL)

You have to have 2/3 of the paper already written to get the grant for the project (med sen, NL)

#### Impact on Research via Length of Funding Period

Another effect of funding on scientific practices was that grants typically are for shorter periods only – typically a couple of years (18 comments). Such limitations can restrict the design of a project and lead to a focus on *“short term deliverables”* (med jun, NL). Only one researcher (nat sen, NL) experienced this effect as positive, and even thought that having such short-term funding could benefit long-term research lines in the end because the expectation of the release of data and new results stimulates you to work harder.

However, most researchers, across countries and disciplines, saw the impact of time-limited funding schemes as a potential danger for doing good science. They expressed that *“it can be difficult to continue a line of research”* (nat sen, NL) and that *“long term research is being prevented” (*nat sen, NL). The latter is a problem because *“big societal problems require long term data”* (nat sen, NL). It was obvious that many researchers considered research done over a long time as highly valuable but endangered by funding practices. In the humanities, some scholars feared short periods of time would not even allow to do any significant research at all. One compared short-term research in the humanities with building “*pre-fab houses, but no cathedrals”* (hum sen, NL).

Most competitive research funding is project based and 3-4-5 years duration. It is highly questionable whether this system adequately supports academic research in the humanities since this research often takes much longer period of times to mature. (hum jun, NL)

Several senior researchers also reported short-term funding as leading to hectic research due to the time pressure, sometimes even leaving some of the gathered data to be un-analysed in the end.

#### Impact on Science via Strategic Grant Applications

Many researchers across countries, seniorities and disciplines mentioned that researchers strategically tailor their research ideas, topics, design, and methods to what they think will likely receive funding (18 comments). Researchers may fit their research to match funder ideas and programs at the expense of own interest and ideas, which can imply impoverishment of science:

Research projects are designed to be funded what might be different to research projects with very innovative and ‘unusual’ ideas (med jun, CH)

Researchers may use previously successful grant applications as templates or restrict the design of a project to the specific funding guidelines. One researcher stated very clearly that *“The first question a researcher will always ask him/herself when writing a grant proposal is: ‘What is the right strategy to get the grant?’”* (nat jun, CH). As a result, one researcher feared decreasing diversity in science:

It makes everyone jump through the same hoops, everyone has to meet roughly the same criteria. In this sense it works against diversity in the Dutch science system. (nat jun, NL)

It can also mean strategically generating income, part of which will be used to fund the ‘real’ research of interest:

sometimes large research proposals may be written to generate income, only a small fraction of which (the spoils) are used to fund basic research that the principal investigators are actually interested in (med jun, NL)

#### Impact on Science by Feeling the Need to Write a ‘Sexy’ Proposal

Across countries, seniorities and disciplines, researchers experienced that supposedly sexy, fashionable, topics and research proposals are more likely to be funded (15 comments):

Funding calls for ‘sexy projects’ (med jun, CH)

However, researchers did not think that these kinds of projects are typically of high scientific value because it does not focus on good science. And though it can have positive effects by building trends, it can have the problematic side effect to reduce diversity in scientific topics, disciplines, methods:

skew/select specific trends, and then everyone jumps on the bandwagon - positive effect is that this can rapidly accelerate a promising direction, negative effect is that it creates bubbles/echo chambers which suck funding away from other directions (since the ultimate pool of money is not infinitely increasing). (nat jun, CH)

### Impact of Grant-writing on Research Time

Another major theme expressed by junior Dutch, senior Dutch, and junior Swiss researchers from all fields was that the constant need to apply for funding (or act as reviewer) is extremely time-intensive and distracts from time spent on ongoing research (28 comments). For junior researchers, this can mean spending a considerable amount of time during a running project on writing an application for the next one. Senior researchers often stated that they do more grant-writing or grant-evaluating than research. This problem is particularly severe if funding rates are low:

Takes up a lot of time and effort that basically goes to waste if the project is not funded - problem especially when, as is the case with NWO, the chances of getting funding are so low. (bad thing) (hum sen, NL)

Some Dutch junior humanities scholars actually doubted the overall value of such a funding system - due to the time investments that are required. The associated administration costs are also thought to be too time consuming by some Swiss researchers, who said that this time could better be spent by doing research.

There were only a handful of positive comments on the effects of funding on time management. These were exclusively given by senior natural science and senior medical researchers across countries. One Swiss medical researcher for example thought that “*competitiveness triggers an environment that stimulates the investment of effort (time, thought, hard work).”* One Dutch senior natural scientist thought that the need to devote some time towards writing grants can provide you with time to do creative thinking.

### The Impact of Publication Pressure on QRPs

Perhaps surprisingly, there were comparatively few comments provided regarding the theme of QRPs. Besides comments mentioning QRPs and sloppy science (16 comments; 5% of total comments), there were also some on outright scientific malpractices (11 comments; 3,5%).

#### Impact on QRPs and Sloppy Science

Interestingly, statements regarding negative effects through publication pressure were made exclusively by junior Swiss researchers in the natural sciences and the humanities, though there was one statement by a junior Dutch humanities scholar as well. This finding stands in direct contrast to my original hypothesis that researchers in a country with a higher funding rate (and thus supposedly less competition) should put a more relaxed focus on publications.

Junior researchers expressed for example the view that the following questionable publication practices are taking place due to the publication pressure: *“splitting research into minimal publishable pieces, self-plagiarism, hasty and not fully careful analyses, etc.”* (hum jun, NL). Another researcher thinks that *“junior researchers may be tempted to write papers with controversial views”* (hum jun, CH), or submit to *“exaggerating impact both in proposal and in publications (overhyping)”* (nat jun, CH).

Several Swiss natural science and humanities juniors emphasized that publication pressure could result in haste versus care. Interestingly, junior researchers then assumed that this is predominately problematic for reviewers who might need to put a lot of effort and time into correcting this. At least some researchers thus apparently thought that sloppy research would eventually get corrected via journal peer reviewing.

One researcher mentioned that publication pressures are not primarily exerted by the funding system but rather by the academic career system:

In my opinion this [rapid publication versus careful analysis] is a problem related to extreme weight given to publication record when academics apply for positions. (nat jun, CH)

On the other hand, several – mostly senior – medical and natural sciences researchers across countries expressed that the publication pressure which the system exerts can also be positive because it ensures that papers are eventually being published.

#### Impact on Research Misconduct

Only four of the in total 53 interviewees commented that competition for funding could result in research misconduct, three of which were either Swiss or Dutch medical senior scientists. One of the Swiss ones for example said that *“the high pressure for success obviously fosters the danger of data fabrication, which is extremely difficult to control”* (med sen, CH). The reasons for misbehaviour, another Swiss said, may be extreme competition amongst PI’s.

However, a Dutch medical scientist commented that if bad practices indeed occur, the problem may have to be viewed in a much broader perspective than funding per se. One would need to consider also “*researcher’s careers, positions, salaries etc”*, because these aspects are judged using the same criteria. Interestingly, the same scientist also admitted that these statements were based on hearsay and not on his/her own experiences. They were thus essentially speculations. It is then interesting to note that another person posited the potential occurrence of severe research misconduct as a question:

if your livelihood depends on it, doesn’t it seem very understandable to tweak the results of your study so to increase the chance of that high impact paper that will help you get your next funding?? (nat jun, NL)

The four other junior Dutch natural scientists in this session all individually reacted to such an (in their eyes) extreme view of unethical behaviour, even though they admitted that scientists may behave in strategic ways and thus do things too sloppily or somewhat biased.

I think ‘cheaters’ is maybe a bit too strong. I would say that the funding system stimulates ‘strategic behaviour’, i.e. behaviour to maximize the quantifiable output of research. (nat jun, NL)

## Discussion and Conclusion

Researchers participating in this study experienced that competition for funding has a drastic effect on scientific practice. While some of these effects are positive, most are perceived as problematic. Those problematic effects, however, were of a quite different nature than what is defined as QRPs in the research integrity literature (Bouter et al., 2016); a mere 9% of the comments provided alluded to such QRPs and malpractices. Publication pressure was experienced to be a more general phenomenon in academia. Contrary to expectations, it was junior researchers in the *low*-competition country which connected funding with publication pressures.

The effects on science that researchers perceived as most important (91% of comments; across all session groups) were directly introduced by funding and were typically of a much broader nature than QRPs, which focus on conducting a study in a correct manner. The underlying mechanism seems to be the following one: funders incentivize researchers to do good science; however, while researchers do appreciate these intentions, those incentives often have negative consequences in practice. For example, funders strive to select the best science by asking for explicit proposals, but the associated peer reviewing process may have the side effect of decreasing diversity. Researchers are also incentivized to create broader impact, to broaden their perspective by collaborating in bigger teams, or to show that their projects are feasible. However, creating impact might bend away from putting sufficient care into the core research; working in teams could turn out to be extremely difficult and diminish individual researcher maturation; and feasibility can result in non-risky predictive research.

I would suggest that many of such intended and unintended aspects fall under the umbrella of the ‘projectification’ of science induced by funding (see also Felt, 2021a). Via shaping science into ‘projects’, funding could lead to at least some science becoming predictable, boring, short-sighted, fashionable and/or overpromising. Researchers are worried that this might make it difficult to do good science that really matters: plunging into the unknown or addressing big issues that need a long-term horizon to mature.

High competition for funding in the Netherlands may have exacerbated such unintended effects of funding (but not QRPs). The Netherlands do not only have a more competitive funding system, but science policy steers research to a much higher degree than in Switzerland (Lepori et al., 2007). Dutch researchers therefore experience less autonomy, for example with regards to research impact, which is often called ‘valorization’ in the Netherlands (de Jong et al., 2016). This effect was visible in my results: Dutch researchers were more vocal and experienced with negative consequences of strong science policies, such as little budget for basic science. Those effects may well overshadow any effects of publication pressure with regards to the Netherlands, which is why I might have found a higher perception of publication pressure among Swiss junior scientists than among Dutch ones. Swiss scientists seemed in comparison much happier with their funding system, which went beyond pure aspects of lower competition; they seemed to value the higher autonomy.

The idea that funding itself results in questionable types of doing science beyond QRPs proper might come as a surprise to some researchers within the research integrity community. One may wonder about the representativeness of my study findings due its relatively low sample size. Can 53 researchers from only two countries really represent the current problems within the science funding system? I think this is possible as my findings are corroborated by the –mostly European– scholarly literature outside research integrity: in fact, it then appears that none of my above findings on how funding shapes science is very novel or surprising.

Scientists have over the years repeatedly pointed out that competing for funding impacts science in often worrying ways (starting as early as in the 1970s, see e.g. Brooks, 1978). There are a whole host of science policy and other studies addressing and discussing the relationship between competitive research funding and scientific practice. Topics include, for example, funder peer review and its biases (Bornmann & Daniel, 2006; Langfeldt, 2006; van den Besselaar & Leydesdorff, 2009), research impact (Wallace & Rafols, 2015; de Jong et al., 2016), and risky versus conservative science (Guthrie et al., 2019; Veugelers et al., 2019; Ayoubi et al., 2021). It is also interesting to note that a large-scale bibliometric study investigating millions of papers recently showed that the rate of disruptive papers is declining, and that one of the possible mechanisms behind this may be “shifting interests” of funders (Park et al., 2023). Some studies have also started making a connection between this literature and the research integrity literature (Conix et al., 2021; Recio-Saucedo et al., 2022). Whether my findings can be generalized beyond what one may call the ‘Western’ world, or possibly even only Europe, however, needs to be explored.

My study is novel in exploring the effects of competitive research funding bottom-up, showing that the current focus on QRPs might misrepresent where the actual problems lie regarding doing good science in connection with funding. This is one of the strengths of ethnographic research: it puts less borders around concepts and topics. Indeed, one of my main findings is that the research integrity field may currently have a too narrow focus on QRPs and misconduct. My findings are corroborated by other studies of a comparable ethnographic kind that have made similar findings with regards to what it would mean to do good science and what currently restricts it (Jerak-Zuiderent et al., 2021), and with regards to the impact of time constraints and projectification (Felt 2021a, b). But are the insights generated by my study still about research integrity per se? Hasn’t it in the end become, as above studies seem to suggest, more about science policy? Shouldn’t we rather strive for a more explicit demarcation of what research integrity actually is (Helgesson & Bülow, 2021)? I would like to note here that other research integrity researchers also already emphasize that there needs to be a shift in focus from individual researcher responsibilities to aspects of the ‘system’ (Bonn & Pinxten, 2019; Bruton et al., 2020; Sørensen et al., 2021). In addition, what is currently understood under research integrity seems to depend already on whom you ask (Davies, 2019; Davies & Lindvig, 2021).

I would suggest that our goal in connection with funding should be to find out what the real problems regarding doing good and valuable science are – and ultimately, what issues funders and other science policy makers should address to improve the situation. Looking at this from several perspectives is certainly valuable. Interestingly, my suggestions are very different from the ones given by Labib et al. (2021) and Roje et al. (2021), who both emphasized that funders should monitor breaches of research integrity and should exert pressure on universities to meet research integrity standards via eligibility for funding. My findings instead indicate that we should shift the focus away from QRPs and narrow research integrity, and rather focus on the unintended consequences of funding that might have a much bigger and more worrisome effect on science. When considering those consequences, implementing more guidelines and putting more pressure on researchers might make matters even worse. I would suggest that funders should, in close conversation with active researchers, instead reflexively re-evaluate how they could enable researchers to do the best possible science. The outcome of such conversations might well be to relax guidelines, monitoring, and expectations rather than tighten them.

## Data Availability

Session reports cannot be made openly available, because even though they are largely anonymized this would not protect the participants’ privacy in a sufficient and responsible manner. For the purpose of this publication, I have made sure to anonymize participants sufficiently in order to preserve privacy. Informed consent from all participants has been received regarding such types of dissemination, but not for making the data openly available.

## Footnotes

1 In the Results, the following abbreviations are being used: med = researcher in a medical field; nat = researcher in a natural science field; hum = researcher in the humanities; jun = junior; sen = senior; NL = researcher currently based in the Netherlands; CH = researcher currently based in Switzerland.
